# Effective therapy with Bismuth-212 labeled macroaggregated albumin in orthotopic mouse breast tumor models

**DOI:** 10.3389/fchem.2023.1204872

**Published:** 2023-05-10

**Authors:** Nathan Kauffman, Satyendra Kumar Singh, James Morrison, Kurt R. Zinn

**Affiliations:** ^1^ Comparative Medicine and Integrative Biology, Institute for Quantitative Health Science and Engineering, Michigan State University, East Lansing, MI, United States; ^2^ Department of Biomedical Engineering, Institute for Quantitative Health Science and Engineering, Michigan State University, East Lansing, MI, United States; ^3^ Advanced Radiology Services, Grand Rapids, MI, United States; ^4^ Departments of Radiology, Biomedical Engineering, Small Animal Clinical Sciences, Institute for Quantitative Health Science and Engineering, Michigan State University, East Lansing, MI, United States

**Keywords:** cancer therapy, macroaggregated albumin (MAA), interventional radiology (IR), Bismuth-212 (Bi-212), alpha-particle, radiopharmaceutical

## Abstract

Intravascularly administered radiation therapy using beta (β-)-emitting radioisotopes has relied on either intravenously injected radiolabeled peptides that target cancer or radiolabeled microspheres that are trapped in the tumor following intra-arterial delivery. More recently, targeted intravenous radiopeptide therapies have explored the use of alpha (α)-particle emitting radioisotopes, but microspheres radiolabeled with α-particle emitters have not yet been studied. Here, FDA-approved macroaggregated albumin (MAA) particles were radiolabeled with Bismuth-212 (Bi-212-MAA) and evaluated using clonogenic and survival assays *in vitro* and using immune-competent mouse models of breast cancer. The *in vivo* biodistribution of Bi-212-MAA was investigated in Balb/c and C57BL/6 mice with 4T1 and EO771 orthotopic breast tumors, respectively. The same orthotopic breast cancer models were used to evaluate the treatment efficacy of Bi-212-MAA. Our results showed that macroaggregated albumin can be stably radiolabeled with Bi-212 and that Bi-212-MAA can deliver significant radiation therapy to reduce the growth and clonogenic potential of 4T1 and EO771 cells *in vitro*. Additionally, Bi-212-MAA treatment upregulated γH2AX and cleaved Caspase-3 expression in 4T1 cells. Biodistribution analyses showed 87–93% of the Bi-212-MAA remained in 4T1 and EO771 tumors 2 and 4 h after injection. Following single-tumor treatments with Bi-212-MAA there was a significant reduction in the growth of both 4T1 and EO771 breast tumors over the 18-day monitoring period. Overall, these findings showed that Bi-212-MAA was stably radiolabeled and inhibited breast cancer growth. Bi-212-MAA is an exciting platform to study α-particle therapy and will be easily translatable to larger animal models and human clinical trials.

## 1 Introduction

Radiation therapy has been essential for cancer management since its inception (Baskar et al., 2012; Ma et al., 2019; Razvi et al., 2019). Many forms of radiation therapy exist, with each having unique properties and indications for use. Two forms of intravascular delivery of radiation therapy are well established in the clinical realm: peptide receptor radionuclide therapy (PRRT) and selective internal radiation therapy (SIRT). PRRT uses radiopeptides targeted against cancer-specific receptors to deliver radiation therapy, while SIRT uses selective intra-arterial (IA) infusion of radioembolics via tumor vasculature to irradiate tumors and stunt blood supply to tumors (Bodei et al., 2011; Arslan et al., 2021). Both methods rely on beta (β-)-emitting isotopes, namely Lutetium-177 (Lu-177) for PRRT and Yttrium-90 (Y-90) for SIRT (Kauffman et al., 2023). β-particles are high-energy electrons that penetrate multiple millimeters into tissue. While the deep penetration allows for bystander treatment of non-targeted cancer cells, healthy cells surrounding the tumor also receive a large radiation dose (Bodei et al., 2011; Ravi Kumar and Hofman, 2020; Morris et al., 2021). This can lead to morbidity and toxicity.

Alpha (α)-particle emitting radionuclides are an exciting alternative to β-emitting radionuclides due to their high linear energy transfer (LET) and shorter range in tissue (Sgouros et al., 2010; Tafreshi et al., 2019; Morris et al., 2021). Alpha (α)-particle emitting nuclides such as Ac-225, Ra-223, At-211, Pb-212, and Bi-213 have been used in preclinical models and clinical trials to treat advanced-stage cancer where limited treatment options were available (Tafreshi et al., 2019; Sgouros et al., 2020). Importantly, there are multiple reports where α-particle emitters were used to treat patients with β-radiation resistant tumors (Kratochwil et al., 2014; Kratochwil et al., 2016; Shi et al., 2022). The large LET of α-particles compared to β-radiation resulted in double-stranded DNA breaks; free radical production was not required to kill cells. This means that radioisotopes emitting α-particles can be efficacious in situations of radioresistance, including hypoxia (Muz et al., 2015). The poor commercial availability of radioisotopes with α-particle emissions has prevented them from becoming well translated into the standard of care (Tafreshi et al., 2019; Sgouros et al., 2020; Strosberg et al., 2023). Novel radiopharmaceutical combinations and delivery strategies can help translate more α-particle emitting radionuclides into the clinic (Kratochwil et al., 2014; Autenrieth et al., 2018; Meredith et al., 2018; Kauffman et al., 2023). Alpha-emitting radioisotopes are becoming more available for preclinical and clinical studies. Examples include Pb-214 and Bi-214 from a Rn-222 generator system (Niowave, Inc.; https://www.niowaveinc.com/index.php/alpha-emitters/)), Ac-225 (from Niowave, Inc.); and future availability of At-211 (from MSU’s Facility of Rare Isotope Beams -FRIB, and Ionetix (https://www.ionetix.com/alpha-therapy/)).

Interventional oncology is a field of medicine that utilizes image guidance to deliver cancer therapies directly into tumors (Degrauwe et al., 2019). The primary options for interventional oncologic therapies include percutaneous ablation and IA embolization. SIRT, also called Yttrium-90 microsphere (Y90) therapy or Trans-Arterial Radio-Embolization (TARE), is used extensively to treat hepatic cancers (Spyridonidis et al., 2020). SIRT can reduce bulky liver tumors and bridge patients to surgical resection or transplantation options. SIRT relies on β-decay to deliver radiation therapy to tumors, which can have adverse effects due to the penetration of β-energy and effects on the nearby liver. Radiation and vascular damage also limit the ability to repeat treatment if there is a residual or recurrent tumor. It is important to retain as much healthy liver as possible, so the use of β-emitters may not be ideal. Additionally, few tumors in extrahepatic tissues have been explored for SIRT due to the penetration range of β-energy and the risk of the intervention blocking the blood supply to normal tissues. An internal locoregional therapy utilizing an α-particle emitting nuclide could improve tumor response and lower toxicity to healthy tissue; currently, none exist.

FDA-approved macroaggregated albumin (MAA) are particles that range in size from 10–70 μm (90% of all particles) and can be labeled with Tc-99m for lung perfusion studies in nuclear medicine or for SIRT pre-treatment planning (Hamami et al., 2009; Amor-Coarasa et al., 2014). MAA is similar in size to the microspheres used for SIRT and predicts deposition of the microspheres after delivery in the same arterial space. Other embolics, such as lipiodol, have been used to test new SIRT delivery platforms, but few exist for MAA (Bozkurt et al., 2016; Bakker et al., 2017). MAA is attractive as a vehicle as it is semi-embolic, meaning it will be lodged into vasculature until it is eventually cleared naturally by the body. Combining the semi-embolic nature of MAA with a short-lived α-particle emitting radionuclide could allow for effective therapy in hepatic tumors but also other tissues more sensitive to radiation and reduction of blood supply. The current studies evaluated MAA as a vehicle for α-particle therapy delivery. Bismuth-212 (Bi-212), which was eluted from a Lead-212 (Pb-212) generator, was selected for binding to MAA due to availability from a generator system, short half-life, and decay scheme with α-particle emission. We also studied Bi-212 labeled MAA (Bi-212-MAA) efficacy in killing and inhibiting the growth of breast cancer cells *in vitro* and *in vivo*.

## 2 Materials and methods

### 2.1 Pb-212 generator elution

The Ra-224/Pb-212 generators (5 mCi) were provided by Oak Ridge National Lab (The isotopes used in this research were supplied by the U.S. Department of Energy Isotope Program, managed by the Office of Isotope R&D and Production). Elution and preparation of the isotope was done similarly to as previously described (Baidoo et al., 2013). The generator was washed with 500 μL of 2 M HCl upon receipt. Every day afterwards, Bi-212 was eluted from the generator with 800 μL of 0.15M KI/0.1 M HCl solution. The eluent was treated with 8 M HNO_3_ and evaporated to dryness 3 times. The dried vials containing the Bi-212 were reconstituted with 100 μL of 0.1 M HNO_3_ for transfer to vials containing 10 μL of 1 M NaOH for neutralization. To confirm the purity of the Bi-212 solution, a small sample from each elution was evaluated with a gamma counter (Wizard2, Perkin Elmer) for the energy peak corresponding to Bi-212 only (600 keV). Bi-212 purity was also confirmed by repeatedly measuring Bi-212 samples over time with a dose calibrator (CRC-25R, Capintec) to measure half-life.

### 2.2 Bi-212-MAA radiolabeling and quality control

FDA-approved MAA kits (Pulmotech) were purchased from Cardinal Health (East Lansing, MI). For radiolabeling MAA with Bi-212, 3 mg of the MAA kit (0.33 mg MAA) was resuspended in 500 μL 1X PBS (pH 7.0) and added to the neutralized Bi-212. The Bi-212-MAA solution was incubated for 10 min at 70 °C with 500 RPM shaking. Bi-212-bound MAA was purified by centrifugation at 1000 *g* for 5 min with the pellet containing the Bi-212 bound MAA and the supernatant containing unbound Bi-212 that was easily removed. The percentage of Bi-212 bound to MAA was determined with instant thin layer chromatography (iTLC) on paper silica gel impregnated strips using 10 mM EDTA in 0.15 M NH_4_OAc as the mobile phase.

### 2.3 Cell lines

4T1 and EO771 cells were purchased from ATCC. Cells were cultured in complete media (RPMI or DMEM with 10% FBS, 1% penicillin/streptomycin and 1% L-glutamine (Thermo Fisher Scientific), respectively) and incubated at 37°C with 5% CO_2_. Cells were subcultured upon reaching 90% confluency and reseeded at 20% confluency.

For generation of stably luciferase expressing cell tines, both 4T1 and EO771 cells were transduced by PLV-10170-pLV-CAG-Firefly luciferase-PGK-Puro plasmid using lentivirus (Cellomics Technology) and luciferase positive clones were selected using puromycin (Thermo Fisher Scientific).

### 2.4 Clonogenic and survival assays

The clonogenic assay was performed similarly to as previously described (Sinha et al., 2021). In this assay, 1 × 10^3^ 4T1 and EO771 Luc + cells were seeded into 6-well plates 12 h prior to treatment. Cells were treated in triplicate with 0, 5, 10 and 20 μCi Bi-212-MAA and incubated for 1 week. Bland, unlabeled MAA particles were used as 0 uCi condition (control group) to match the number of particles used for the highest treated condition (20 μCi). For IVIS imaging and quantification, cells had media removed and replaced with 150 μg/mL luciferin diluted in complete media. Cells were imaged using IVIS Lumina bioluminescence imaging (BLI) with autoexposure setting. Data were analyzed using ROI and radiance (photon/second). After imaging, colonies were stained with crystal violet and manually counted for the total number of colonies per well. Colonies were considered for clusters with greater than 50 cells.

For the survival assay, 5 × 10^4^ 4T1 and EO771 Luc + cells were seeded into a 24-well plate. 12 h later, cells were treated with 0, 2.5, 5, 10, or 20 μCi of Bi-212-MAA and incubated for 48 h. Bland, unlabeled MAA was again used as the 0 μCi condition (Control group), with the number of particles matching those used in the 20 μCi condition. IVIS imaging and quantification were done identically to the clonogenic assay as described.

### 2.5 Western blot analysis

Total protein was extracted by lysing the 4T1 cells using RIPA buffer (Thermo Fisher Scientific) with 1X proteinase inhibitor cocktail added (Abcam) and incubated at 4°C for 30 min and vortexed 45 s every 10 min. Cell lysates were centrifuged at 30,000 g for 30 min at 4°C, and the supernatants were collected and stored at −80°C for further use. Protein concentration was determined using the Bradford protein assay method. Protein samples were denatured by adding 4 x Laemmli sample buffer (BioRad) and heating 95°C for 5 min. Further, 40 μg of proteins were run on 4–15% SDS-PAGE gels. Proteins were transferred onto the nitrocellulose membrane, followed by blocking with a blocking buffer for 1 h at room temperature. Next, membranes were incubated with diluted primary antibody (1:1000; Supplementary Table S1) in a blocking buffer at 4°C with gentle shaking. Membranes were washed three times with 1X TBST buffer and further probed with polyclonal anti-rabbit or anti-mouse IgG secondary antibodies conjugated to horseradish peroxidase (HRP) and incubated at room temperature for 1 h. After washing three times with 1X TBST buffer, enhanced chemiluminescence (ECL) solution (Thermo Fisher Scientific) on the top of the membrane, and protein bands were visualized using Chemidoc (BioRad).

### 2.6 Animals

All animals were purchased from Charles River Laboratory. All animals used in the experiments were of Balb/c and C57BL/6 background (female) and 8 weeks of age. The animal studies were approved by the Institutional Animal Care and Use Committee (IACUC), and all animal experiments were conducted according to IACUC guidelines.

### 2.7 Bi-212-MAA imaging and biodistribution in Balb/c and C57BL/6 mice

Balb/c and C57BL/6 mice (8 weeks old) were implanted with 1 × 10^5^ 4T1 and EO771 cells (respectively) in the left 4th mammary gland using a 50:50 mixture of Matrigel (Thermo Fisher Scientific) and PBS in a total volume of 50 uL. After 10 and 11 days (4T1 and EO771, respectively), 4 mice with breast tumors were injected intratumorally (IT) with either 11 μCi (4T1) or 7 μCi (EO771) of Bi-212-MAA in 20 μL of 0.9% sterile saline using 25-gauge integrated needle syringes with zero dead volume. The 4 mice plus 1 additional untreated mouse with a breast tumor were then imaged 2 h later using Cherenkov luminescence imaging (CLI) on IVIS Spectrum for radiation localization. After imaging, all 5 mice were injected with luciferin (3 mg) intraperitoneally and imaged 5 min later using BLI for tumor localization. Mice were sacrificed after the imaging session and had organs removed for gamma counting. Injection data and gamma counted data were all decay corrected to the counting start time. Mice in the 4-h group underwent biodistribution without an imaging session and were counted in the same fashion as the 2-h group with proper decay correction.

### 2.8 Orthotopic mammary tumor treatment with Bi-212-MAA

Balb/c and C57BL/6 mice (8 weeks) were implanted with 1 × 10^5^ 4T1 and EO771 Luc + cells (respectively) in the left 4^th^ mammary gland. After 7- and 8-days post-implantation, 4T1 and EO771 tumors, respectively, were injected IT with Bi-212-MAA or vehicle control (bland, unlabeled MAA). 4T1 mice received either 25 or 50 uCi of Bi-212-MAA or control, while EO771 mice received either 50 or 100 uCi Bi-212-MAA or control suspended in 20 μL of 0.9% sterile saline using 25-gauge integrated needle syringes with zero dead volume. Mice were then tracked for tumor growth using digital caliper measurement. All groups were euthanized once the tumor size reached 2 cm in length in any group. Tumor volume was calculated using the equation (longest diameter x ((shortest diameter/2)^2)).

### 2.9 Statistical analysis

Statistical analysis was performed using GraphPad. One-way analysis of variance (ANOVA) with Dunnett’s test was used to compare the experimental groups to the control group. Data are reported as mean ± standard error of the mean (SEM). *p*-values <0.05 were considered statistically significant.

## 3 Results

### 3.1 Efficacy of MAA radiolabeling with Bi-212

**FIGURE 1:**
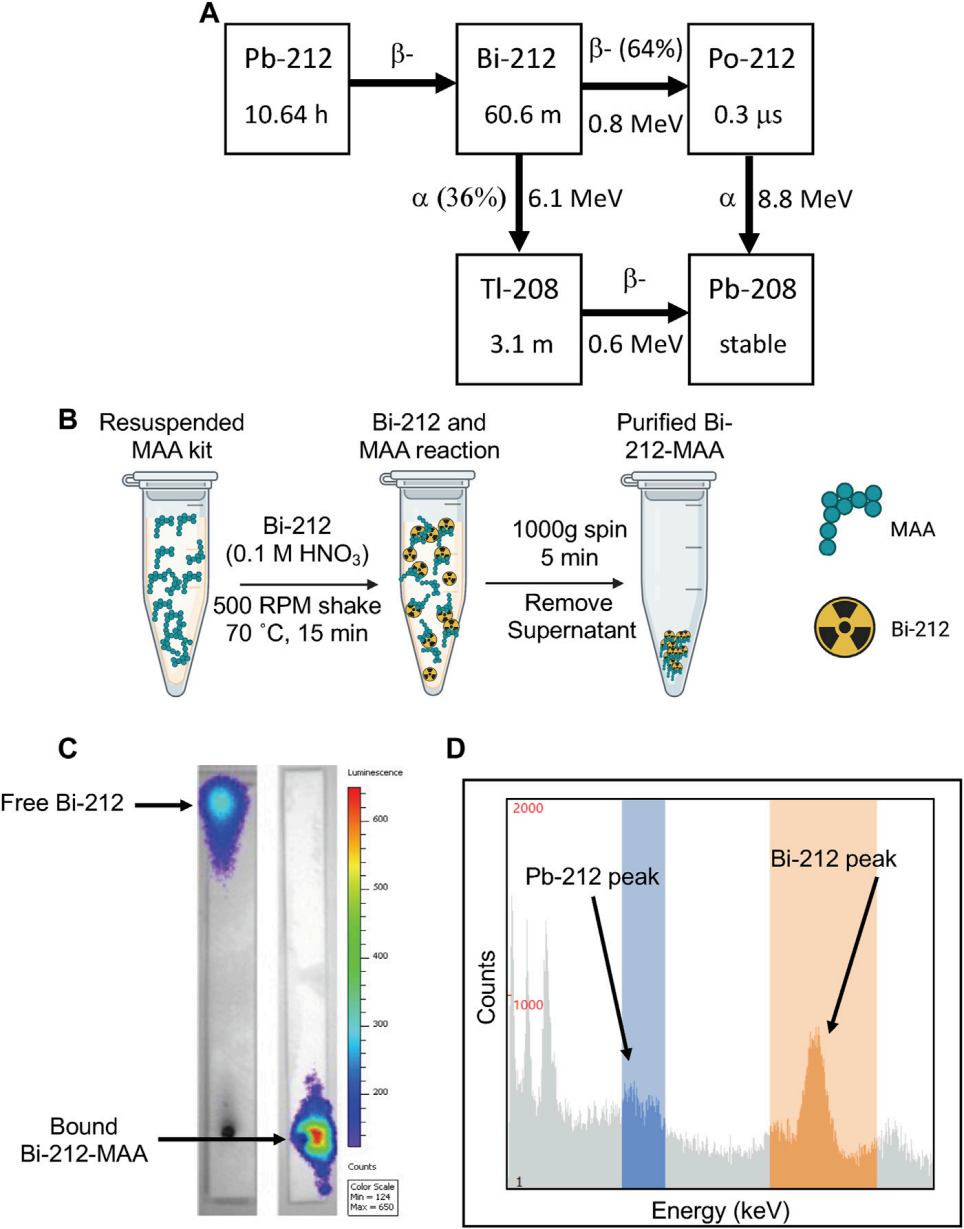
Overview of radiolabeling and quality control protocols for Bi-212 MAA assays. **(A)** Decay scheme for Pb-212 available from Ra-224 parent. Bi-212 has a shorter half-life and avoids the beta decay emission arising from Pb-212. **(B)** Graphical overview of Bi-212-MAA radiolabeling procedure, highlighting the speed and simplicity of the procedure. **(C)** Representative CLI picture of developed iTLC strips of either pure Bi-212 (left) and purified Bi-212-MAA (right) using IVIS machine. Free Bi-212 moved to the top of the strip while Bi-212 bound to MAA remained at the bottom. **(D)** Representative gamma counting window present on Wizard gamma counter during a counting protocol. Bi-212 has a gamma peak around 600 keV (orange window) while Pb-212 has a peak at 200 keV (blue window) that can be used to discern the radionuclides in a sample.

Bi-212 was successfully eluted from the Ra-224 column (Pb-212 generator) and used for all assays. The decay scheme for Pb-212 is shown in Figure 1. Bi-212 or short-lived Po-212 (0.3 μs) had the critical α-particle emissions and excludes the β-emission from Pb-212 decay (Figure 1A). The commercially available MAA kits were radiolabeled using simple methods, as shown in Figure 1B. This allows for quick use of the Bi-212-MAA and limits total decay of the activity. Many radiolabelings were performed throughout the life of the generator for the different assays. For example, on a day a week after the Pb-212 generator was received, the pure Bi-212 (484 ± 4 μCi) was added to 3 mg MAA kit (n = 3), incubated, and purified. The resulting Bi-212-MAA was 240 ± 1 μCi, indicating an efficiency of 50%. The MAA kits are 10% MAA and contain 4 million MAA particles. Approximately 667,000 MAA particles were contained within 3 mg of MAA kit, and it was assumed that all were retained after purification. This resulted in a specific activity of 240/0.3 μCi/mg of MAA particles, or 0.8 μCi/μg. The Bi-212-MAA used for treatment studies were done when the generator had higher activity with the highest being approximately 20 μCi/μg MAA particles. iTLC strips were used to determine purity and the strips were counted with either the dose calibrator or gamma counter depending on activity. IVIS-based CLI of an example iTLC strip is shown in Figure 1C, with the EDTA mobile phase able to push free Bi-212 to the top of the strip while Bi-212 tightly bound to MAA remains at the bottom. To confirm the purity of the Bi-212 initial elution and the final Bi-212-MAA product, a sample was removed and counted on the gamma counter. Visualization of the peak at around 600 keV (orange window) and lack of a peak at 200 keV (blue window) confirms the presence of Bi-212 only (Figure 1D). Only Bi-212-MAA conjugate with purity >90% were used for *in vitro* and *in vivo* studies.

### 3.2 Bi-212-MAA delivered radiation therapy and inhibited clonogenic potential, and killed breast cancer *in vitro*

**FIGURE 2:**
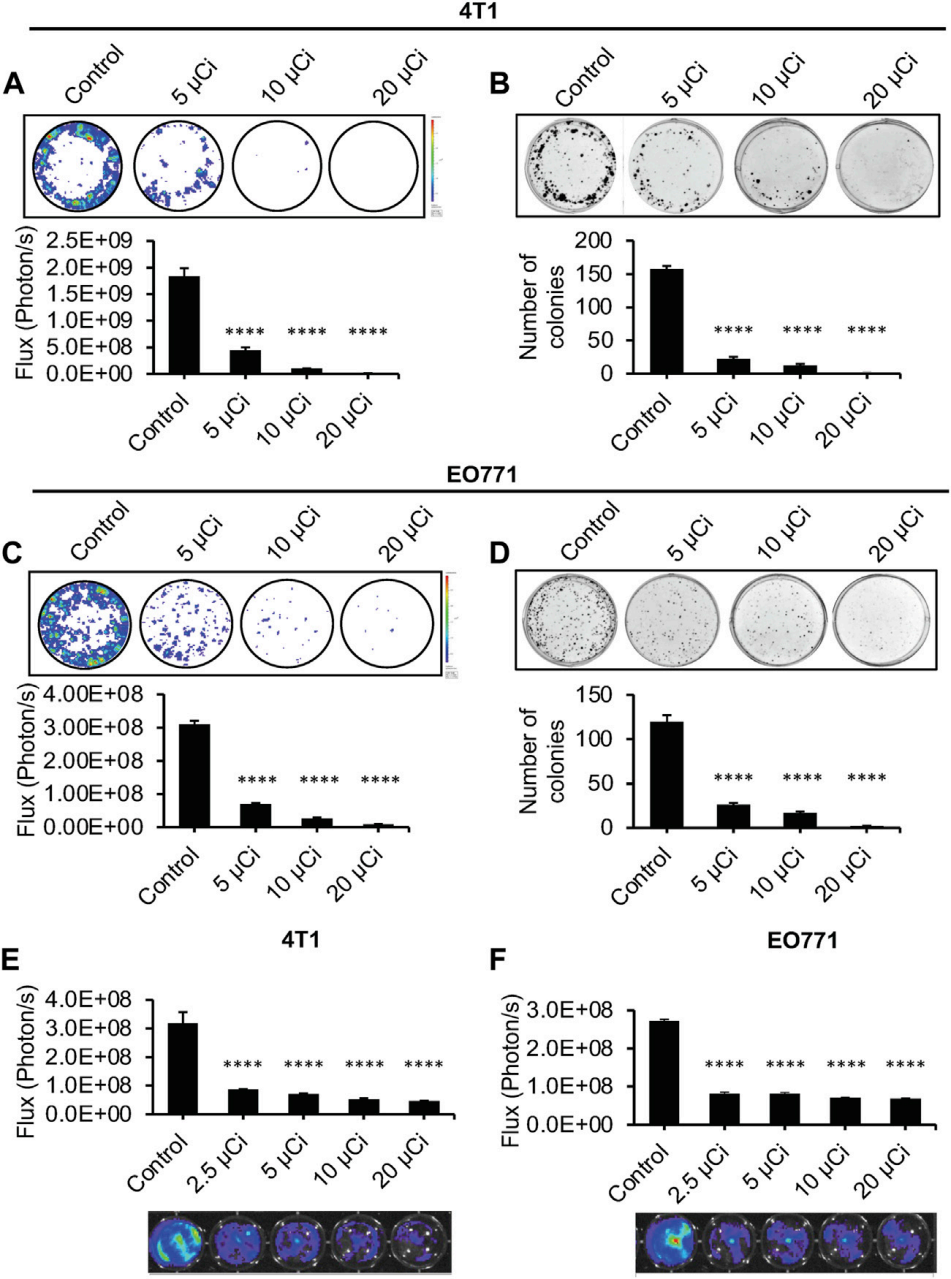
Effective killing of 4T1 and EO771 cells *in vitro* with Bi-212-MAA. **(A–D)** Representative images of BLI and crystal violet staining of the clonogenic assay for 4T1 **(A,B)** and EO771 **(C,D)** showed a specific dose response in cancer cell reproductive death (n = 3). **(E,F)** Representative images of BLI of the survival assay 48 h after treatment with Bi-212-MAA showed dose specific reduction in 4T1 **(E)** and EO771 **(F)** cell growth (n = 4). ****, *p* < 0.0001 compared to control.

MAA particles spread out evenly in solution and sink, allowing for treatment of monolayer cell culture (Supplementary Figure S1). Previously, the treatment efficacy of radiotherapy was evaluated by clonogenic and survival assays for various cancer cells, with the clonogenic assay measuring the colony formation capacity of individual cancer cells and their ability to replicate (Hu et al., 2016). In this study, we used Bi-212-MAA to treat 1 × 10^3^ 4T1 and EO771 breast cancer cells in a 6-well plate format to assess radiation therapy effectiveness on cancer cell clonogenicity. Increased levels of Bi-212-MAA led to significantly less total colony formation in both 4T1 and EO771 cells (Figures 2A–D). This was first assessed using BLI, which shows the total amount of signal in the plate and not the individual colony number (Figures 2A,C). Afterward, cells were stained with crystal violet, and the number of colonies was manually counted (Figures 2B,D). All treatment groups showed significantly decreased BLI signal when compared to bland MAA control for both 4T1 and EO771 cells (Figures 2A,C). After staining and counting, all treatment groups showed significantly decreased colony formation when compared to bland MAA control in both 4T1 and EO771 cells (Figures 2B,D).

These results correlated well with a short-term survival assay (Figures 2E,F). Cells in a survival assay are grown at a higher confluency and thus are reactive to paracrine functions and other communications from neighboring cancer cells. They are also able to reach their exponential phase of growth more quickly compared to the clonogenic assay. Bi-212-MAA was added to cells in an increasing dose to identify the sensitive dose range. After 48 h, there was a significant decrease in the total number of cells for both 4T1 and EO771 cells in all treatment conditions compared to the bland MAA control (Figures 2E,F). There was a significant difference in all treatment groups compared to the control, indicating that even the lowest dose of 2.5 μCi was sufficient to kill or prevent reproduction in most cells.

### 3.3 Bi-212-MAA upregulated DNA damage and cell death markers in 4T1 cells

**FIGURE 3:**
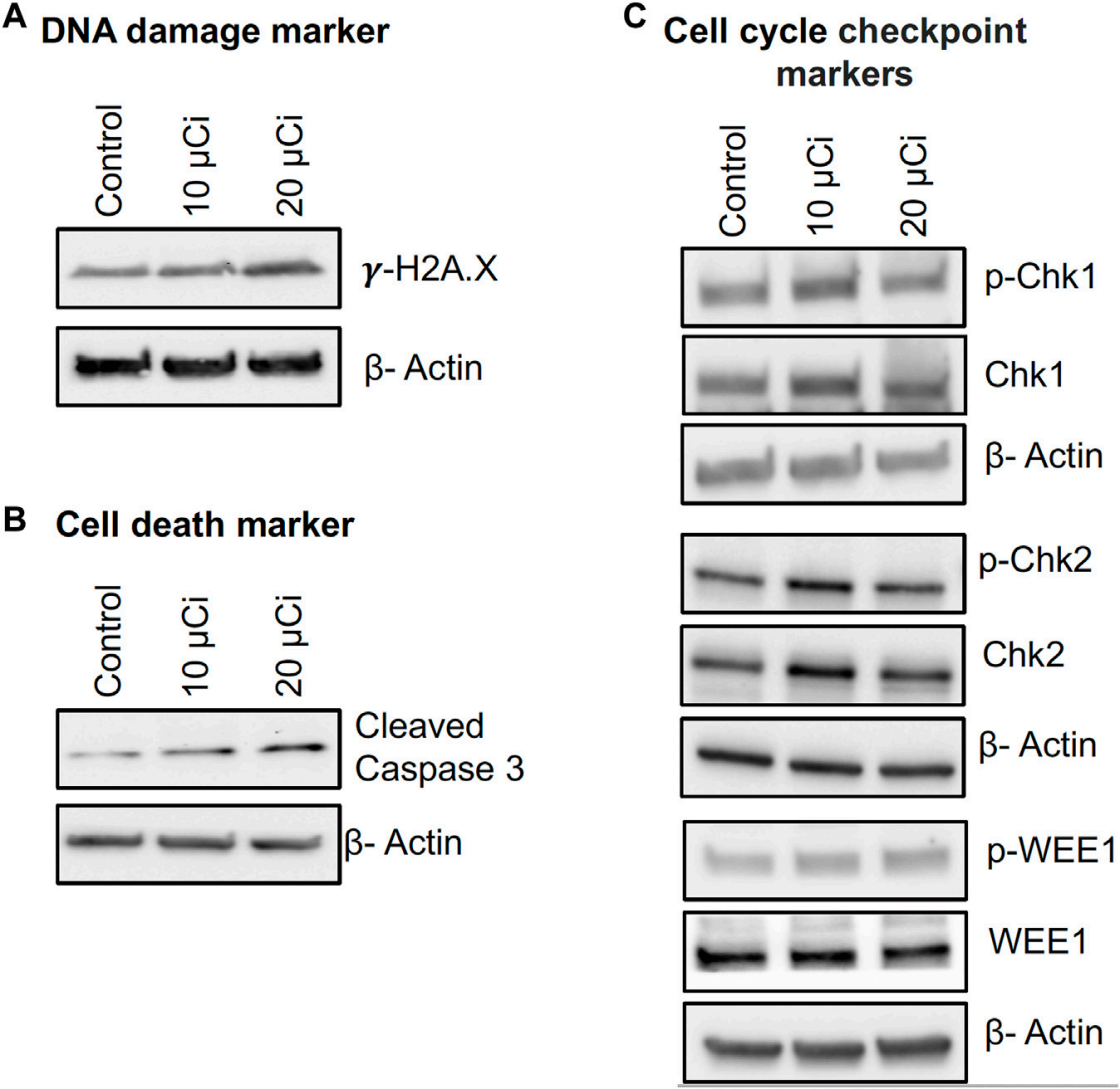
Bi-212-MAA treatment induces DNA damage, apoptosis, and cell cycle pathways in mouse breast monolayer cancer cell culture. **(A–C)** Western blots showing Bi-212-MAA treatment results in induction of DNA damage **(A)** and apoptosis markers **(B)** and comparable effect on cell cycle checkpoint markers **(C)** in higher Bi-212 activity group compared to control.

The clonogenic and survival assays showed that Bi-212-MAA was effectively killing breast cancer cells and preventing cell growth. Therefore, to find out the downstream molecular markers involved in cell death, a DNA damage marker and a cell death marker were evaluated. H2AX phosphorylation (γH2AX) is a well-known marker of an early cellular response to DNA double-strand breaks, and Caspase 3 is a key molecule involved in cell death (Chen et al., 2022; Widjaja et al., 2023). In this study, western blot analysis showed that Bi-212-MAA treatment induced H2AX phosphorylation in 4T1-treated cells (Figure 3A). Further, a higher expression of cleaved Caspase-3 was found in Bi-212-MAA treated 4T1 cells compared to control cells (Figure 3B).

Next, the expression of cell cycle checkpoint markers was studied to elucidate if 4T1 cells could effectively initiate DNA damage repair response (Chan Wah Hak et al., 2022). The results showed that at a lower dose of Bi-212-MAA, there was increased activation of cell cycle checkpoint markers Chk1, Chk2, and Wee1; however, at higher dose levels, Chk1, Chk2, and Wee1 activation were comparable to control (Figure 3C). These findings suggest that Bi-212-MAA treatment to 4T1 cells caused DNA damage and killed breast cancer cells without activating cell cycle checkpoint markers at a higher dose which could avoid cell senescence and potential radioresistance.

### 3.4 Bi-212-MAA was safely delivered to orthotopic breast tumors in mice via intratumoral injection and significantly decreased tumor growth

**FIGURE 4:**
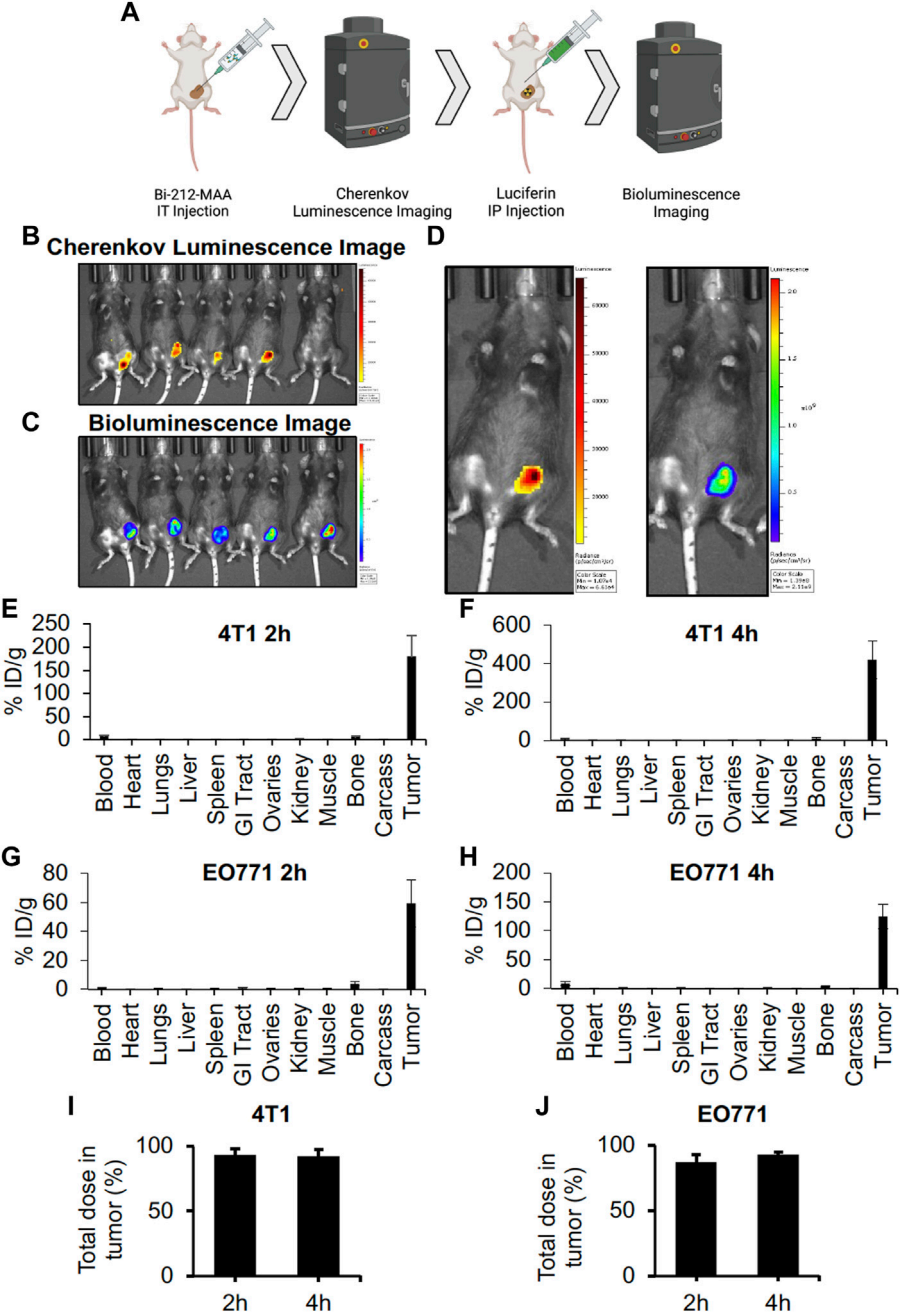
Intratumorally injected Bi-212-MAA was stably retained in the tumoral space. **(A)** Mice used for the Bi-212-MAA imaging and biodistribution were treated as indicated. **(B,C)** Bi-212-MAA remained within tumor as shown by the CLI **(B)** picture and tumor tissue was confirmed with BLI **(C)** of Luc + EO771 cells comprising the tumor. **(D)** Blow up of representative CLI and BLI images of the same mouse confirmed overlay of both data sets. **(E–H)** Complete biodistributions indicated most of the injected Bi-212-MAA remains in the tumor at 2 and 4 h in both the 4T1 **(E,F)** and EO771 **(G,H)** tumors (n = 4). Some radiation signal in the blood and bone potentially indicated free nuclide while minimal signal in lungs indicated minimal escape of Bi-212-MAA from the tumor. **(I,J)** The percentage of the total dose within the 4T1 **(I)** and EO771 **(J)** tumors indicated minimal leakage of Bi-212-MAA into healthy organs (n = 4).

Mice are too small to use IA delivery of drugs into tumor vasculature, so direct IT injection was needed for delivery of radioembolics into tumor tissue. It is well known that large particles can be retained in solid tumors after injection (Rosemurgy et al., 2008; Bakker et al., 2017; Morsink et al., 2022). Mice with orthotopic EO771 tumor were injected IT and imaged 2 h later. CLI showed that Bi-212-MAA stayed within the tumor space (Figures 4A–D). This is indicated by the CLI data (Bi-212-MAA radiation) margins being within the BLI data (Luc + EO771 tumor cells) margins.

At 2- and 4-h post-injection, greater than 90% of the total injected dose remained within the tumor for both 4T1 and EO771 (Figures 4F–J). A full biodistribution of the mice revealed minimal leakage out of the tumor and into the lungs, which was the expected destination for any Bi-212-MAA that entered post-tumoral venules (Figures 4F–J).

**FIGURE 5:**
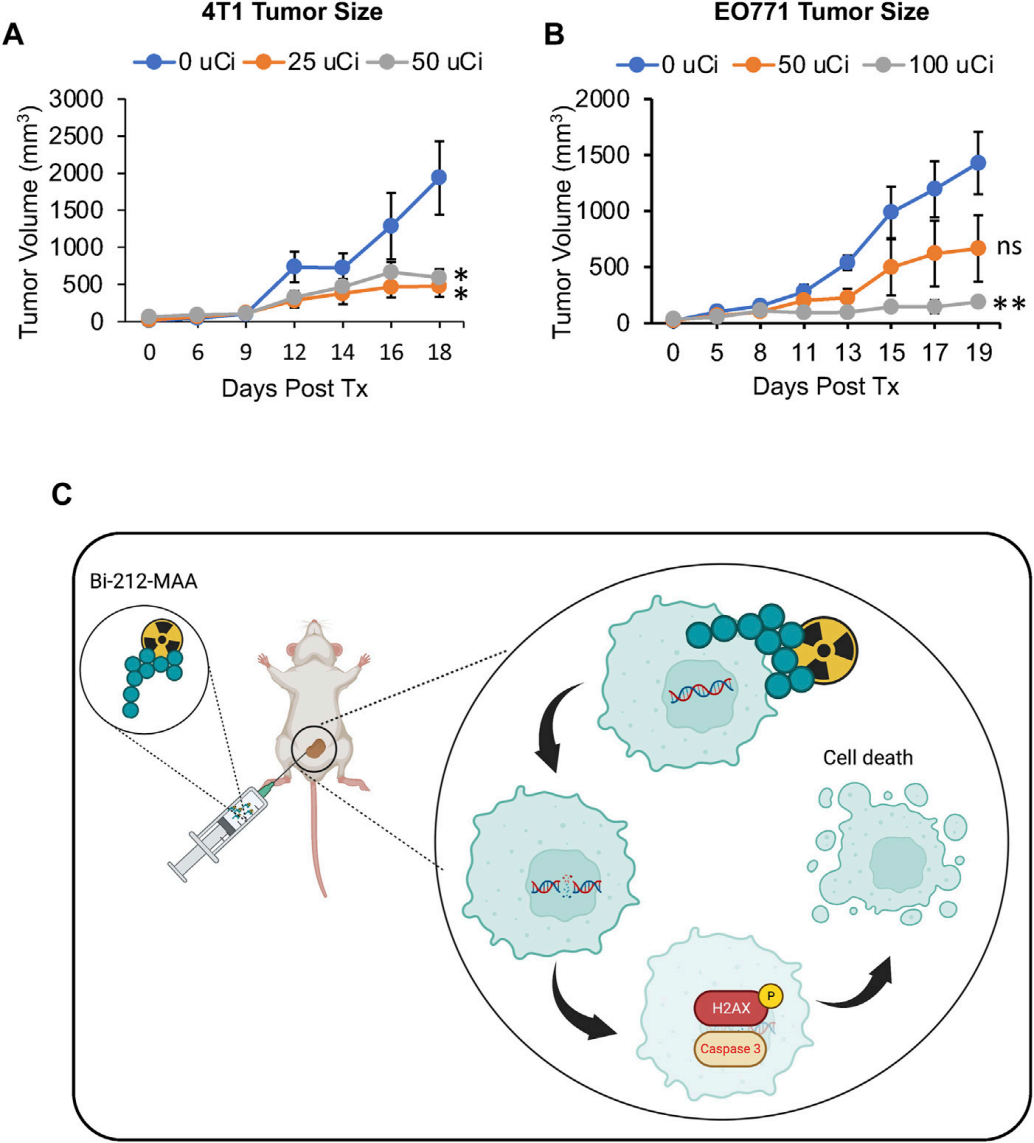
Intratumoral delivery of Bi-212-MAA prevented orthotopic breast tumor growth. **(A,B).** Tumor volume growth was decreased in both 4T1 **(A)** and EO771 **(B)** orthotopic breast cancer models (n = 4). **(C)** Schematic picture showing potential mechanism involve in Bi-212-MAA therapy mediated breast cancer cell death. ns, not significant; *, *p* < 0.05; **, *p* < 0.01 compared to control.

Further, to evaluate the effectiveness of Bi-212-MAA in killing breast cancer cells *in vivo*, 4T1 and EO771 tumors in Balb/c and C57BL/6 mice, respectively, were treated with Bi-212-MAA by injecting IT. The Bi-212-MAA caused a significant reduction in total growth compared to bland MAA-treated controls in both 4T1 and EO771 tumors (Figures 5A,B). These results indicated that IT injection was a viable route for Bi-212-MAA delivery. Additionally, these results indicated that Bi-212-MAA effectively reduced breast cancer cells growth in the mouse orthotopic models. Overall, our findings suggest that Bi-212-MAA can kill breast cancer cells and reduce tumor growth by regulating cell death (Caspase 3) and DNA damage (γH2AX) marker (Figure 5C).

## 4 Discussion

A standard, commercially available MAA kit was rapidly labeled with Bi-212 and easily purified. The purification method was easier compared to other methods, that can take additional time, and the product can be lost during the process. The decay scheme of Pb-212 and pure elution of Bi-212 allowed for simple labeling and quantification of Bi-212-MAA. The process can be improved even further by using clinical grade generators when they become available.

Due to its size and density, MAA sank in solution and thus contacted cells in monolayer cell culture when added. With the short range of α-particles, it was unknown whether the MAA would bring the Bi-212 close enough to cells to deliver an effective therapy. However, the results clearly indicate that the Bi-212-MAA was an effective therapeutic against mouse breast cancer cells *in vitro* in both the clonogenic and survival assays. Importantly, we can perform further *in vitro* studies to study the molecular effects of α-particle radiation on cancer cells.

It was found that classic markers of apoptosis and general DNA-damage response markers were elevated with increasing levels of Bi-212-MAA. Further, no change was seen in markers of radioresistance, specifically proteins involved in cell cycle checkpoint control. It is known that increased radiation dose can lead to cell cycle arrest and cell survival, making it critical to find a sensitive dose (Chan Wah Hak et al., 2022; Chen et al., 2022; Widjaja et al., 2023). Alpha (α)-particle therapy works through direct double-stranded DNA breaks, thus leaving the cell with very few options for resistance and survival. Other forms of radiation, including photon and β-, rely on single-stranded breaks and the generation of free radicals for cell destruction. These are much easier to overcome compared to double-stranded breaks and allow ample opportunity for cancer cell escape.

The large size of MAA allows it to remain in tumor when delivered IT as seen with our biodistribution studies. The 2- and 4-h time points both showed high levels of MAA retention. These time points represented 75% and 94% of the total decay of Bi-212, respectively. The low dose present in healthy tissues shows that MAA stayed lodged within the tumor and that the radiopharmaceutical was stable and not releasing Bi-212. Further time points were not needed as greater than 90% of the dose was still present in the tumor at the 94% decay point. The biodistribution data were corroborated using CLI and BLI.

Both 4T1 and EO771 tumors showed a response to IT delivery of Bi-212-MAA. This is an impressive result since the MAA is likely not distributed uniformly throughout the tumor and is therefore not leading to a complete tumor tissue dose. Although the CLI data shows retention of Bi-212-MAA in tumors, it does not cover the margins of the BLI data, indicating a smaller area of localization at the injection site. In an IA delivery model, the distribution would be much more uniform. Additionally, the expected range of α-particles from Bi-212 decay would span multiple cell diameters, allowing for a local crossfire effect in the tumor without causing toxicity to surround healthy parenchyma. Mice vessels were too small to utilize image guidance; however, there was precedence for direct injection of drugs into tumors to study therapy. With the short half-life of Bi-212, Bi-212-MAA only needed to remain within the tumor for a few hours to deliver a large radiation dose to tumors, which explains the success of our therapy studies.

Due to Bi-212-MAA being agnostic to tumor receptor presence or density, it can be used to explore the effects of α-particles on any solid tumor model. Critically, it was recently found that fractionated external beam radiation therapy combined with immunotherapy led to an abscopal response in mice (Vanpouille-Box et al., 2017). Systemic immune response against cancer induced by radiation therapy would be an excellent treatment outcome and is a worthy goal sought. Bi-212-MAA can be used to explore its own effects on pan-cancer immunogenicity *in vitro* because of its agnostic targeting, similar to external beam radiation. Further, the short half-life of Bi-212 allows for a similar fractionated therapy schedule compared to the external beam, while long-lived isotopes such as Y-90 will continually dose the tumor once delivered (Aicher et al., 2022).

Bi-212-MAA also represents an easily translatable α-particle emitting radiopharmaceutical. As MAA and Ra-224/Pb-212 generators are already used in clinical trials, Bi-212-MAA has great promise for translation to human studies as an IA delivered drug. SIRT is a widely used treatment option for reducing hepatic tumor size and bridging patients to either surgical or transplantation options. The recent success of SIRT in the prostate highlights the potential for radioembolization in tissues outside of the liver (Mouli et al., 2021). The breast represents another tissue that may be amenable to IA therapies (Zhang et al., 2013). Bi-212-MAA could be used not only to improve results in hepatic tumors but also safely explore new treatment options for prostate and breast cancers.

In this study, we were able to effectively radiolabel MAA with the short-lived α-particle emitter Bi-212. Additionally, Bi-212-MAA was successfully used to inhibit mouse breast cancer growth in monolayer cell culture and in orthotopic tumor models. Bi-212-MAA represents a widely applicable platform for studying the effects of α-particle therapy on cancer cells, including molecular mechanisms of radioresistance and immunogenicity. Further, Bi-212-MAA is uniquely poised to be translated into clinical trials due to all reagents and its required delivery method being FDA approved.

## Data Availability

The original contributions presented in the study are included in the article/Supplementary Material, further inquiries can be directed to the corresponding author.
