# Supplementary material for: Effective therapy with Bismuth-212 labeled macroaggregated albumin in orthotopic mouse breast tumor models
Source: Front Chem. 2023 May 10;11:1204872. doi: 10.3389/fchem.2023.1204872 (PMC10206259; doi:10.3389/fchem.2023.1204872)
Supplement: Supplementary file 1 [file Table1.DOCX]

| **Antibody** | **Manufacturer** | **Catalogue number** |
| --- | --- | --- |
| γH2AX (Ser139) | Cell Signaling Technology | 9718 |
| Cleaved Caspase 3 | Cell Signaling Technology | 9664 |
| Phospho-Chk1 (Ser345) | Cell Signaling Technology | 2348 |
| Chk1 | Cell Signaling Technology | 2360 |
| Phospho-Chk2 (Ser379) | Thermo Fisher Scientific | PA5-64776 |
| Chk2 | Cell Signaling Technology | 2662 |
| Phospho-Wee1 (Ser642) | Thermo Fisher Scientific | MA5-14806 |
| Wee1 | Abcam | ab273016 |
| β-Actin | Cell Signaling Technology | 4970 |

**Supplementary Table 1.** List of antibodies used for western blot
