# Supplementary material for: Effective therapy with Bismuth-212 labeled macroaggregated albumin in orthotopic mouse breast tumor models
Source: Front Chem. 2023 May 10;11:1204872. doi: 10.3389/fchem.2023.1204872 (PMC10206259; doi:10.3389/fchem.2023.1204872)
Supplement: Supplementary file 2 [file DataSheet1.PDF]

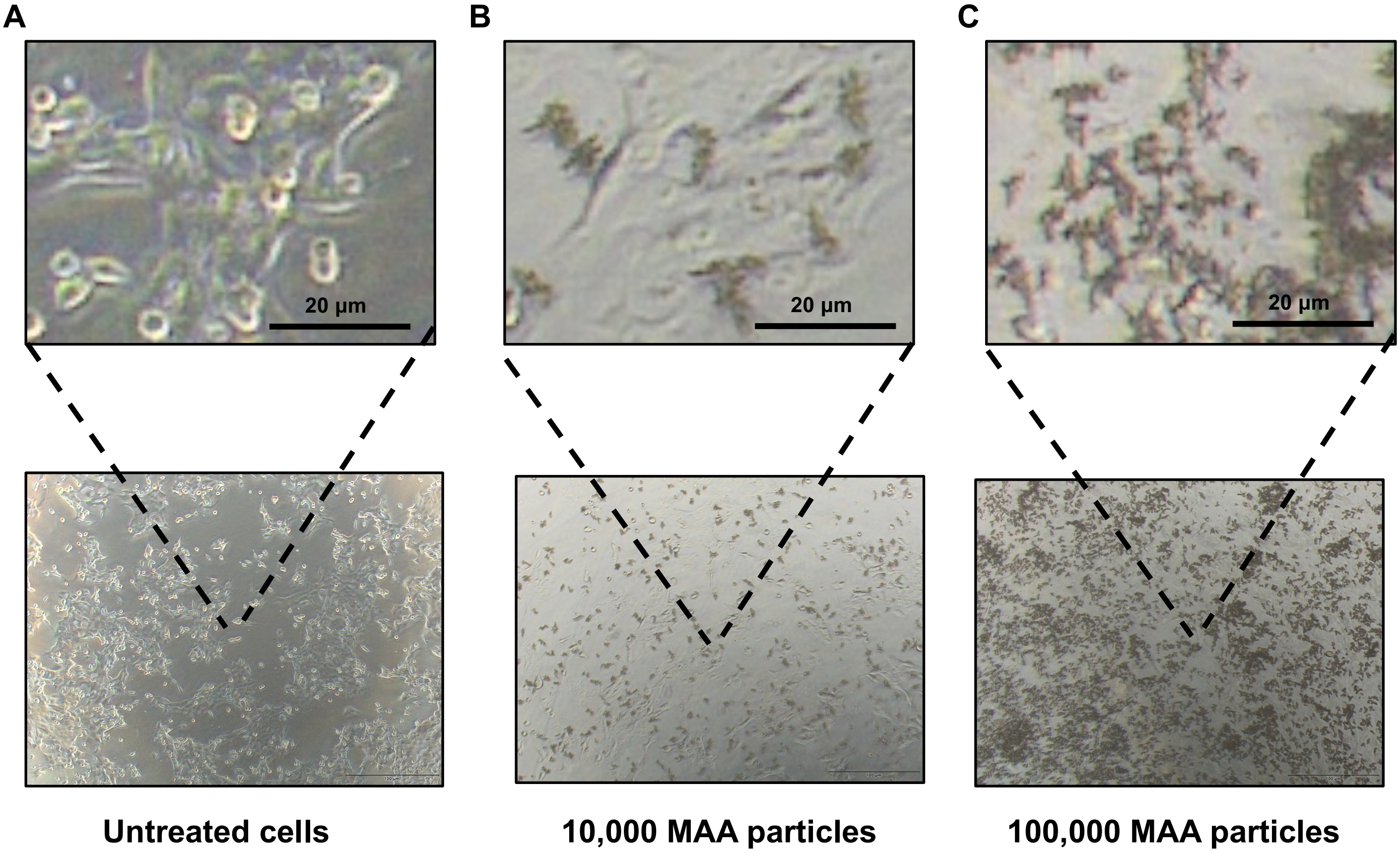

Supplementary Figure 1 | MAA distributes evenly throughout cell culture plates. (A-C) 4T1 cancer cells in a 24-well plate **(A)** are well covered by MAA regardless of lower **(B)** or higher **(C)** amounts of MAA particles.
